# High feasibility and safety of office-based transurethral laser ablation for recurrent non-muscle invasive bladder cancer: a single-center experience

**DOI:** 10.3389/fonc.2026.1683009

**Published:** 2026-04-20

**Authors:** Zoran Zimak, Dominic Vidovic, Tomislav Kulis, Dinko Hauptman, Tvrtko Hudolin, Mirko Bakula, Zeljko Kastelan

**Affiliations:** 1 Department of Urology, University Hospital Centre Zagreb, Zagreb, Croatia; 2 Sveuciliste u Zagrebu Medicinski fakultet, Zagreb, Croatia; 3 Hrvatska akademija znanosti i umjetnosti, Zagreb, Croatia

**Keywords:** holmium-YAG laser, non-muscle invasive bladder cancer, outpatient surgery, recurrence, transurethral laser ablation

## Abstract

**Objective:**

To evaluate the feasibility, safety, and early oncological outcomes of office-based transurethral laser ablation (TULA) for recurrent non-muscle invasive bladder cancer (NMIBC).

**Methods:**

We retrospectively reviewed 53 patients undergoing their first TULA for recurrent NMIBC at the University Hospital Centre Zagreb from March 2024 to February 2025. Inclusion criteria comprised histologically confirmed NMIBC recurrence suitable for outpatient laser ablation. Exclusion criteria included carcinoma *in situ*, muscle-invasive disease, prior intravesical therapy, or tumors >3 cm. Procedures were performed under local anesthesia using a holmium-YAG laser. Clinical, procedural, and oncological outcomes were analyzed, with predictors of recurrence assessed via multivariate logistic regression.

**Results:**

The median patient age was 68 years, and 54.7% were male. Most tumors were pTa (94.3%) and low-grade (92.5%); 90.6% measured <1 cm. TULA was completed successfully in 94.3% of cases, with minimal peri-procedural complications (1.9%). At 3 months, the recurrence rate was 38.8%. Univariate analysis linked recurrence with older age, higher Charlson Comorbidity Index, and male gender. In multivariate analysis, male gender remained the only independent predictor (aOR 4.29; p=0.034). No patient progressed to muscle-invasive disease.

**Conclusions:**

Office-based TULA is a highly feasible, safe, and cost-effective alternative for selected patients with small recurrent low-grade NMIBC, offering significant procedural and economic advantages over transurethral resection. Although early recurrence rates are notable in this high-risk cohort, oncological safety appears acceptable. Male gender may predict early recurrence, warranting further investigation in larger prospective studies.

## 1 Introduction

Bladder cancer poses a significant public health burden, both globally and in Croatia. It currently ranks as the ninth most common cancer worldwide by incidence, while in Croatia, it is the fifth most frequent malignancy in men and among the top ten in women (1, 2).

The recurrent nature of non-muscle invasive bladder cancer (NMIBC), which constitutes approximately 75% of cases, places a heavy load on both patients and healthcare systems (3). This burden manifests as the need for repeated procedures and lifelong surveillance due to recurrence rates that can reach up to 77% (4). Consequently, treatment and follow-up strategies are guided by the European Association of Urology (EAU) risk stratification.

While transurethral resection of the bladder tumor (TURBT) remains the gold standard for management, its primary drawback is the requirement for spinal or general anesthesia (3). This is a key consideration for the typically elderly NMIBC patient population, who often present with significant comorbidities. The procedure is particularly burdensome for patients, and costly for the healthcare system, when managing superficial, low-grade recurrent tumors (5, 6).

This clinical scenario highlights the need for a safe and oncologically sound outpatient treatment modality for a select group of patients. Transurethral laser ablation (TULA) has emerged as one such method, performed under local anesthesia in a day-case setting. Indications for TULA typically include small (e.g., <1–3 cm), recurrent, papillary tumors of low-grade malignancy (pTa LG) (7–9).

Given the potential benefits of TULA in reducing the treatment burden of recurrent NMIBC, it is crucial to evaluate its application in a local clinical context. The aim of this study is to report our initial experience with office-based TULA for the treatment of NMIBC at the Department of Urology, University Hospital Centre Zagreb, focusing on the feasibility, safety, procedural characteristics, and early oncological outcomes.

## 2 Materials and methods

### 2.1 Study design and patient population

We conducted a retrospective review of patients treated at the Urology Clinic, University Hospital Centre Zagreb. The study cohort was composed of patients who underwent their first TULA procedure for recurrent NMIBC between March 2024 and February 2025.

Inclusion criteria were: age >18 years, patient’s ability to understand and provide informed consent, a history of at least one prior TURBT with histological confirmation of NMIBC, and a cystoscopically identified recurrence consistent with disease suitable for office-based ablation.

Exclusion criteria were: any previous histological confirmation of carcinoma *in situ* (Tis), clinical suspicion of muscle-invasive bladder cancer (MIBC), a history of intravesical BCG or chemotherapy, tumor characteristics unsuitable for TULA (size >3 cm, extensive multifocality, or inaccessible location), or patient refusal.

During the study period, a total of 56 patients underwent their first TULA procedure. After applying the exclusion criteria, primarily due to a history of intravesical therapy (n=3), the final study cohort consisted of 53 patients. The TULA procedure was not performed during the same cystoscopic session in which the recurrence was diagnosed. All patients first underwent a diagnostic cystoscopy to identify the recurrence. Following this, they were scheduled for the office-based TULA as a separate procedure. The time interval between the diagnostic cystoscopy and the TULA procedure was typically within 2 to 3 weeks. The procedure was performed using a holmium-YAG laser. Energy settings were adjusted according to tumor size, with standard initial settings of 0.8 J and an 8 Hz frequency. A 365-micrometer laser fiber was predominantly used for ablation. Regarding discontinuation of antiplatelet or anticoagulant therapy our approach was differentiated based on the type of medication: Antiplatelet therapy (e.g., acetylsalicylic acid) was not discontinued for the procedure; Anticoagulant therapy was temporarily discontinued based on the agent. Direct Oral Anticoagulants (DOACs) were stopped 3 days prior to the procedure, while Warfarin (Martefarin) was stopped 5 days prior with LMWH bridging when indicated.

### 2.2 Data collection and variables

Data were collected from electronic health records, including patient demographics, comorbidities, smoking status, and detailed tumor characteristics from endoscopic reports. Prior to analysis, data accuracy was verified. For patients with more than one tumor, the diameter of the largest lesion was recorded for analysis. Multifocality was defined as the presence of tumors in two or more distinct locations within the bladder. Comorbidities were quantified using the Charlson Comorbidity Index (CCI), which was modified to exclude points for the diagnosis of bladder cancer.

### 2.3 Statistical analysis

All statistical analyses were performed using JASP software (Version 0.19.3; JASP Team, 2024). Categorical variables were presented as frequencies and percentages, and compared using Fisher’s exact test. Continuous variables were presented as medians with interquartile ranges (IQR) and compared using the Mann-Whitney U test. A multivariate logistic regression model was constructed to identify independent predictors of recurrence. A two-tailed P value of < 0.05 was considered statistically significant.

## 3 Results

### 3.1 Patient and tumor characteristics

A total of 53 patients were included in the final analysis. The baseline patient and tumor characteristics are summarized in **Table 1**.

**Table 1:** Baseline patient and tumor characteristics.

Characteristic		N (%) or median (IQR)
Age, years		68 (14)
BMI, kg/m²		25.7 (4.2)
CCI score		3 (3)
Number of prior TURBTs		2 (2)
Gender	Male	29 (54.7%)
	Female	24 (45.3%)
Smoking status	Yes	26 (49.1%)
	No	23 (43.4%)
	Unknown	4 (7.5%)
T stage	PUNLMP	1 (1.9%)
	Ta	50 (94.3%)
	T1	2 (3.8%)
Grade	Low Grade	49 (92.5%)
	High Grade	3 (5.7%)
	N/A	1 (1.9%)
Tumor size, cm	<1	48 (90.6%)
	1-2	4 (7.5%)
	2-3	1 (1.9%)
Tumor location	Multifocal	15 (28.3%)
	Posterior wall	15 (28.3%)
	Bladder dome	7 (13.2%)
	Right lateral wall	5 (9.4%)
	Trigone	3 (5.7%)
	Left ureteral orifice	3 (5.7%)
	Bladder neck	2 (3.8%)
	Left lateral wall	1 (1.9%)
	Anterior wall	1 (1.9%)
	Right ureteral orifice	1 (1.9%)
Number of tumors	1	23 (43.4%)
	2	6 (11.3%)
	≥3	24 (45.3%)
BMI category	Underweight	1 (1.9%)
	Normal weight	16 (30.2%)
	Overweight	25 (47.2%)
	Obesity	5 (9.4%)
	Missing	6 (11.3%)

The median patient age was 68 years (IQR 14), and the majority of patients were male (29, 54.7%). The median CCI score was 3 (IQR 3).

Most tumors were low-grade (49, 92.5%) and stage pTa (50, 94.3%). Regarding other tumor characteristics, the vast majority of lesions were small, with 48 (90.6%) tumors measuring less than 1 cm in diameter. Most patients presented with either a solitary tumor (23, 43.4%) or three or more tumors (24, 45.3%). Multifocal tumors were observed in 15 patients (28.3%). The posterior wall was the most frequent single location, also occurring in 15 patients (28.3%).

### 3.2 Safety and efficacy outcomes

The primary procedural and safety outcomes are presented in **Table 2**. The TULA procedure was successfully completed in 50 out of 53 cases (94.3%, 95% CI 84.2% – 98.8%). The operative time ranged from 5 to 20 minutes, depending on tumor size, number and location. The procedure could not be completed in three cases (5.7%). In one case, the procedure was aborted due to intraoperative findings of tumor progression in size and number compared to the initial cystoscopy. In the two remaining cases, the procedure was abandoned because the tumor size and location were deemed unsuitable for complete laser ablation in an office setting. All three unsuccessful procedures were attempted on tumors larger than 1 cm.

**Table 2:** Safety and efficacy outcomes.

Variable	Level	Counts	Total	Proportion	p	95% CI for proportion	
						Lower	Upper
Successful procedure	No	3	53	0.057	<.001	0.012	0.157
	Yes	50	53	0.943	<.001	0.842	0.988
Peri-procedural complications	Yes	1	53	0.019	<.001	4.776×10-4	0.101
	No	52	53	0.981	<.001	0.899	1.000
30-day complications	Yes	1	53	0.019	<.001	4.776×10-4	0.101
	No	52	53	0.981	<.001	0.899	1.000
3-month recurrence rate	Yes	19	49	0.388	0.152	0.252	0.538
	No	30	49	0.612	0.152	0.462	0.748

Proportions tested against value: 0.5.

Peri-procedural complications occurred in 1 patient (1.9%, 95% CI 95% <0.001 – 10.1%). This event was a case of mild hematuria that was successfully managed by endoscopic coagulation of a minor bleeding site. The overall 30-day complication rate was also 1.9% (1, 95% CI <0.001 – 10.1%), representing a different patient who experienced mild hematuria that resolved with conservative management.

### 3.3 Oncological outcomes and predictors of recurrence

The 3-month disease recurrence rate was 38.8% (19, 95% CI 25,2% – 53.8%).

While 53 patients were initially included, 3-month recurrence data were available for 49. This difference is because one patient was lost to follow-up, and three patients were excluded from the study because they were not suitable for TULA and were referred for TURBT.

**Figure 1:**
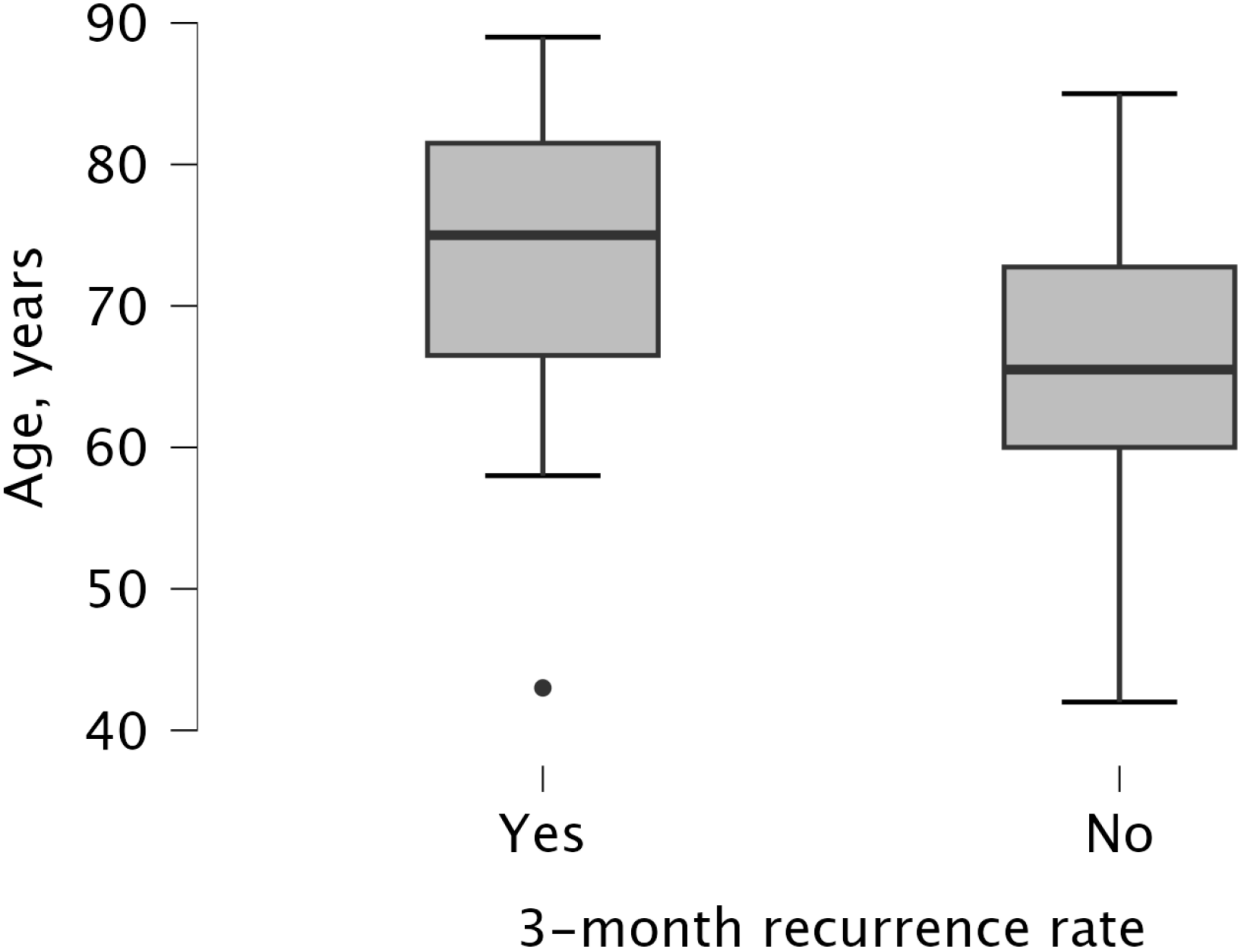
Recurrence rates at three months by patient age.

**Figure 2:**
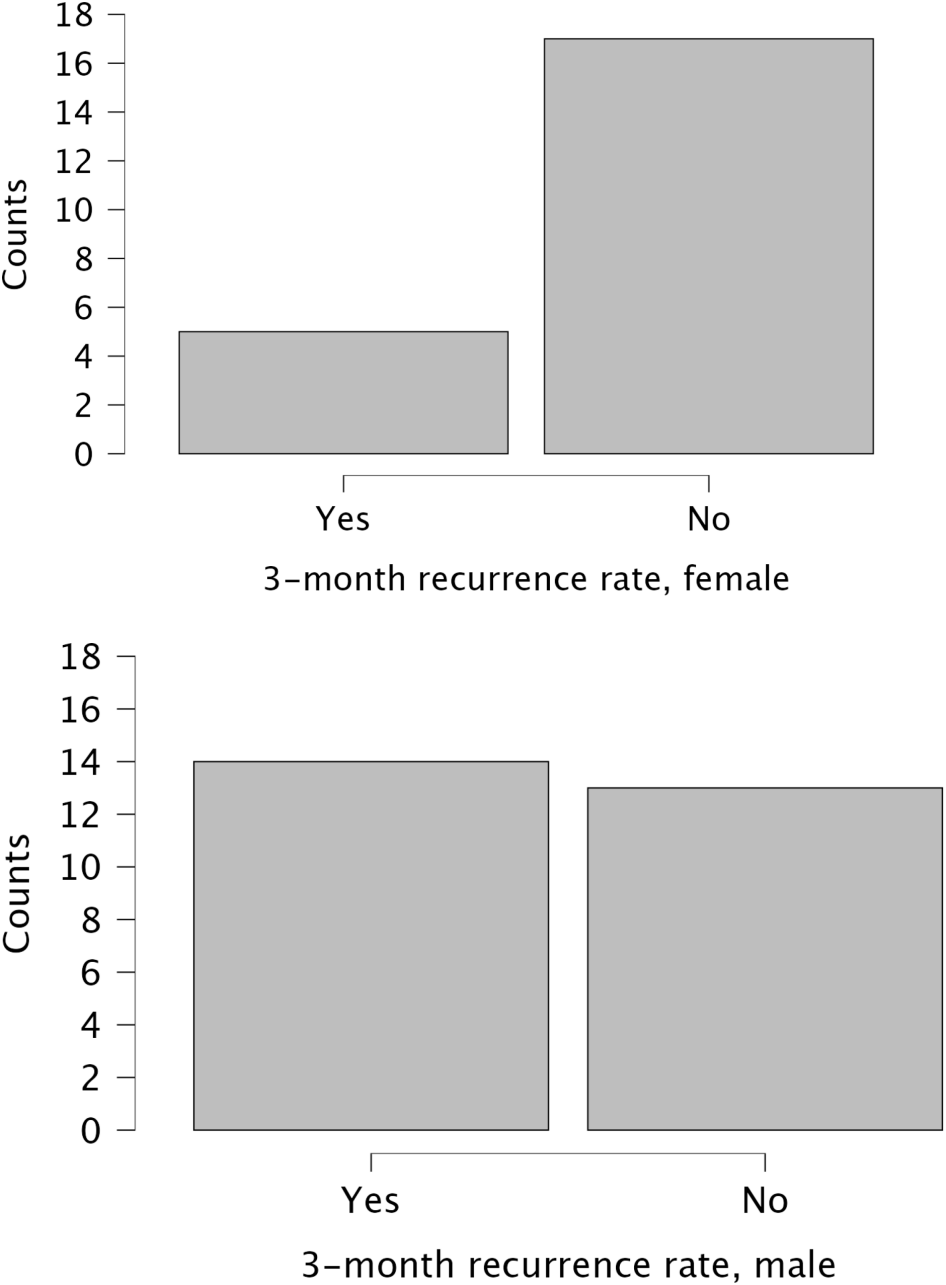
Recurrence rates at three months stratified by gender.

A univariate analysis was performed to identify risk factors for recurrence (**Table 3**). Statistically significant associations were found for older age (median 75 vs. 66 years, p=0.025), a higher CCI score (median 4 vs. 3, p=0.025), and male gender (recurrence rate 51.9% vs. 22.7% for females, p=0.045). A clinically notable, though not statistically significant, trend was observed for tumor multifocality, which was associated with a higher recurrence rate (58.3%) compared to unifocal disease (32.4%, p=0.173). No significant association was found for tumor grade, T stage, size, number of tumors, smoking status, or the number of prior TURBTs. The difference in age between recurrence groups and the recurrence rates by gender are visualized in **Figures 1** and **2**, respectively.

**Table 3:** Analysis of risk factors for disease recurrence.

Univariate and multivariate analysis of risk factors for 3-month disease recurrence					
Characteristic	No recurrence (N = 30)	Recurrence (N = 19)	Univariate p-value	Adjusted OR¹ (95% CI)	Multivariate p-value
Age. years [median (IQR)]	66 (13)	75 (15)	0.025	0.94 (0.88 – 1.01)	0.080
Gender [n (% Male)]	13 (43.3%)	14 (73.7%)	0.045	4.29 (1.11 – 16.50)	0.034
CCI score [median (IQR)]	3 (2)	4 (4)	0.025	—	—
Smoking status [n (% Yes)]	15 (51.7%)	10 (62.5%)	0.544	—	—
Number of prior TURBTs [n (% 2 or more)]	18 (60.0%)	13 (68.4%)	0.762	—	—
Number of tumors [n (% 3 or more)]	13 (43.3%)	8 (42.1%)	1.000	—	—
Tumor size [n (% ≥1 cm)]	2 (6.7%)	0 (0.0%)	0.515	—	—
Tumor location [n (% Multifocal)]	5 (16.7%)	7 (36.8%)	0.173	0.57 (0.11-2.84)	0.492

¹Adjusted for age. gender. and tumor multifocality in a multivariate logistic regression model.

IQR, interquartile range; OR, odds ratio; CI, confidence interval.

Continuous variables compared using Mann-Whitney U test; categorical variables compared using Fisher’s exact test.

To assess the independent effect of these factors, a multivariate logistic regression model was constructed including age, gender, and tumor multifocality. In the final model, male gender remained a statistically significant independent predictor of disease recurrence (aOR = 4.29; 95% CI: 1.11 – 16.50, p=0.034). The effects of age (p=0.080) and tumor multifocality (p=0.492) were not statistically significant in the adjusted model.

### 3.4 Analysis of recurrence characteristics

The characteristics of the 19 recurrence events are detailed in **Table 4**. Recurrence at a new bladder location was observed in 9 (47.4%) of cases.

**Table 4:** Characteristics of tumor recurrence.

Recurrence location	Frequency	Percent	Valid percent
Same as primary	10	52.6	52.6
New location	9	47.4	47.4
Missing	0	0.0	
Total	19	100.0	
Recurrence T stage	Frequency	Percent	Valid Percent
Same as primary	3	42.9	60.0
Progression	2	28.6	40.0
Missing	2	28.6	
Total	7	100.0	
Recurrence Grade	Frequency	Percent	Valid Percent
Same as primary	4	57.1	80.0
Change in grade	1	14.3	20.0
Missing	2	28.6	
Total	7	100.0	

Management of these recurrences consisted of a planned repeat TURBT for 7 patients, while a repeat TULA was planned for 12 other patients. At the time of this analysis, the TURBT had been performed in 6 of the 7 planned cases. Histopathological analysis was available for 5 of these 6 cases. Among these 5 patients, progression in T stage (from pTa to pTis) was noted in 2 cases, and progression to a higher tumor grade was observed in 1 case. Importantly, no patient in this cohort showed progression to muscle-invasive disease.

## 4 Discussion

This study reports on our initial experience with office-based transurethral laser ablation (TULA) for a selected cohort of 53 patients with recurrent non-muscle invasive bladder cancer. The procedure proved to be highly feasible and safe in an outpatient setting under local anesthesia, with a success rate of 94.3% and a low 30-day complication rate of 1.9%. The 3-month recurrence rate was 38.8%. Notably, in our multivariate analysis designed to identify predictors for recurrence, male gender was found to be the sole independent risk factor.

The feasibility of the procedure in an office-based setting was high. The few procedural failures were due to intraoperatively identified tumor characteristics: including size, location, or progression since the last cystoscopy that were deemed unsuitable for complete office-based ablation. The procedure’s safety profile was excellent. The overall 30-day complication rate was minimal; the adverse events observed were minor (Clavien-Dindo grade I-II) and were managed without requiring hospitalization. Large-scale data shows that over 95% of patients have no complications with mild hematuria being the most frequent adverse event. The incidence of post-procedural urinary tract infections was only 0.2% (8). In our series, we observed no infectious complications and did not employ routine antibiotic prophylaxis. Data strongly supports the safety of TULA as an outpatient procedure for selected patients (7–9). In 2005 Sangar et al. estimated that the annual cost of managing bladder cancer was £55.39 million with an average of £8349.20 per patient in the UK. According to these data in 5 year follow up of each bladder cancer case approximately 60% of the cost fall in the category of the low grade TURBT (10). Beyond the clinical benefits, this outpatient approach also offers a substantial economic advantage. At our institution, the average cost of a traditional TURBT is approximately €550, and requires hospitalization, while TULA is strictly an outpatient procedure costing around €150, representing a cost reduction of nearly 73% per procedure.

The observed 3-month recurrence rate of 38.8% is a central finding of our study. This rate is notably higher than the 10–20% short-term recurrence rates typically reported for low-risk NMIBC after a standard TURBT (11). This difference may be explained by our patient cohort, which exclusively consisted of patients with recurrent disease, a group inherently at a higher risk for subsequent recurrence than patients with primary tumors. However, it is crucial to note that despite this higher recurrence rate, our sub-analysis of the recurrence characteristics revealed a favorable oncological profile. Among the patients with available histology, progression in stage or grade was infrequent, and most importantly, no patient progressed to muscle-invasive disease. Long-term studies on TULA show a similar pattern. One study found that about 60% of patients were recurrence-free after one year, but that number fell to roughly 32% after five years (8). Other reports also show recurrence rates over 50% (9). Even though those studies saw slower recurrences than our 3-month results, the overall trend is high. Importantly, even if tumors return, they rarely progress. One major study reported that 86.8% of patients did not progress over five years (8). This supports the oncological safety of this approach for the selected population.

Perhaps the most intriguing finding of our study was the identification of male gender as a strong and independent predictor of early recurrence in the multivariate model (aOR = 4.29). This is notable, as gender is not consistently reported as a major independent risk factor in larger, more heterogeneous NMIBC cohorts. This finding could be influenced by unmeasured confounding variables. For example, a large study using a Cox regression model found that age was the only independent predictor of recurrence (8). Traditionally, we blamed smoking for these gender differences. However, studies show that even after adjusting for smoking status, men still face a higher risk. This suggests that smoking is not the only reason. Biology, specifically the immune system and sex hormones, likely plays a major role. The androgen receptor (AR) seems to be a key driver here. In preclinical models, the androgen receptor has been identified as an important driver of urothelial carcinogenesis and disease progression. AR signaling may also influence the effectiveness of intravesical treatments. In bladder cancer models, AR activation appears to reduce the efficacy of BCG, while blocking the receptor may increase sensitivity to the therapy. These biological factors suggest that the male bladder environment may be naturally more prone to early recurrence (12–14).

When comparing TULA to other deintensified treatment strategies, such as active surveillance (AS) and chemoablation, we acknowledge AS as a valid strategy for a highly select group of patients, aimed at reducing overtreatment. However, AS is not without significant drawbacks. It demands crucial patient compliance for a strict follow up and its application is largely limited to a clinical trial context at the moment (15). Furthermore, AS carries a notable psychological burden of watchful waiting. In this context, we position TULA as a proactive and practical alternative. It offers definitive management of visible lesions in a single office visit. This approach resolves both the lesion and the patient’s anxiety, while still completely avoiding the anesthesia, hospitalization, and significantly higher costs of formal TURBT, as highlighted by our cost analysis. Chemoablation is another promising non-surgical path, with novel formulations like MMC-gel showing high complete response rates (16). However, this approach has two key limitations compared to TULA. First, this approach requires multiple instillations, resulting in substantially higher overall costs compared to the single-procedure treatment with TULA. Secondly, chemoablation carries a risk of more significant side effects. For instance high-efficacy gels can cause treatment-limiting complications, such as ureteral strictures. In contrast, TULA is a physical ablation, completed in a single visit. As our study showed, it is exceptionally safe. We had a very low complication rate of only 1.9%, which was just one case of minor, easily managed hematuria (Clavien-Dindo I-II). This safety profile is a major advantage compared to the potential complications of chemical treatments.

Our study has several limitations. Its retrospective design is susceptible to selection bias and incomplete data. The modest sample size limits the statistical power of our analyses and contributes to the wide confidence intervals observed in the regression model; therefore, the findings should be considered exploratory. Additionally, the short follow-up period provides insight only into early recurrence patterns. Finally, the absence of a control group treated with TURBT prevents any definitive conclusions regarding the comparative efficacy of TULA.

In conclusion, our initial experience suggests that office-based TULA is a safe and feasible treatment for selected patients with small, recurrent low-risk NMIBC. The procedure has a very low complication rate, acceptable early oncological outcomes and a significantly lower cost in comparison with the golden standard of TURBT. We identified male gender as a strong independent predictor of recurrence, a finding that highlights the need for further prospective studies with more detailed data on confounding factors and longer follow-up to confirm these results.

## Data Availability

The raw data supporting the conclusions of this article will be made available by the authors, without undue reservation.
